# Impact of early childhood infection on child development and school performance: a population-based study

**DOI:** 10.1136/jech-2024-222040

**Published:** 2024-08-31

**Authors:** Wen-Qiang He, Hannah Catherine Moore, Jessica E Miller, David P Burgner, Olivia Swann, Samantha J Lain, Natasha Nassar

**Affiliations:** 1 Child Population and Translational Health Research, Children's Hospital Westmead Clinical School, The University of Sydney, Sydney, New South Wales, Australia; 2 Menzies Centre for Health Policy and Economics, Faculty of Medicine and Health, University of Sydney, Camperdown, NSW, Australia; 3 Wesfarmers Centre of Vaccines and Infectious Diseases, Telethon Kids Institute, The University of Western Australia, Perth, Perth, Australia; 4 School of Population Health, Curtin University, Bentley, Western Australia, Australia; 5 Murdoch Children’s Research Institute, The Royal Children's Hospital Melbourne, Melbourne, Victoria, Australia; 6 Department of Paediatrics, The University of Melbourne, Melbourne, Victoria, Australia; 7 Department of Paediatrics, Monash University, Clayton, Victoria, Australia; 8 Department of Child Life and Health, The University of Edinburgh, Edinburgh, UK; 9 Centre for Medical Informatics, Usher Institute of Population Health Sciences and Informatics, The University of Edinburgh, Edinburgh, UK

**Keywords:** INFECTIONS, CHILD HEALTH, EPIDEMIOLOGY, PEDIATRICS

## Abstract

**Background:**

Childhood infection might be associated with adverse child development and neurocognitive outcomes, but the results have been inconsistent.

**Methods:**

Two population-based record-linkage cohorts of all singleton children born at term in New South Wales, Australia, from 2001 to 2014, were set up and followed up to 2019 for developmental outcome (N=276 454) and school performance (N=644 291). The primary outcome was developmentally high risk (DHR) at age 4–6 years and numeracy and reading below the national minimum standard at age 7–9 years. Cox regression was used to assess the association of childhood infection ascertained from hospital records with each outcome adjusting for maternal, birth and child characteristics, and sensitivity analyses were conducted assessing E-values and sibling analysis for discordant exposure.

**Results:**

A higher proportion of children with an infection-related hospitalisation were DHR (10.9% vs 8.7%) and had numeracy (3.7% vs 2.7%) and reading results (4.3% vs 3.1%) below the national minimum standard, compared with those without infection-related hospitalisation. In the multivariable analysis, children with infection-related hospitalisation were more likely to be DHR (adjusted HR 1.12, 95% CI 1.08 to 1.15) and have numeracy (adjusted HR 1.22, 95% CI 1.18 to 1.26) and reading results (adjusted HR 1.16, 95% CI 1.12 to 1.20) below the national minimum standard. However, these results may be impacted by unmeasured confounding, based on E-values of 1.48–1.74, and minimal association with education outcome was found in the sibling analysis.

**Conclusions:**

Infection-related hospitalisation was modestly associated with adverse child development and school performance, but the association may be explained by shared familial factors, particularly in those with most socioeconomic disadvantages.

**WHAT IS ALREADY KNOWN ON THIS TOPIC**

**WHAT THIS STUDY ADDS**

- Childhood infection requiring hospitalisation in the first 4 years of life was associated with a modest increased risk of developmental vulnerability and poorer literacy and numeracy scores from population-based cohorts. Findings may be overestimated due to unmeasured confounding, with socioeconomic disadvantage having a stronger effect than infection on outcomes and no significant associations following sibling cohort analysis except for numeracy.

**HOW THIS STUDY MIGHT AFFECT RESEARCH, PRACTICE OR POLICY**

- Childhood infection requiring hospitalisation might not be associated with an increased risk of developmental vulnerability and poorer literacy and numeracy outcomes. Future studies are needed to validate these findings, particularly applying sibling design and to also assess the effect of specific infections with a relatively small sample size.

## Introduction

 The first 5 years of life are a critical period for cognitive, physical and emotional development.^1^ Preschool children who do not attain their age-appropriate developmental milestones are at increased risk of subsequent academic and socioemotional problems.^2^ Childhood infection has been identified as a potential factor linked to suboptimal childhood development.^3^ While some pathogens could impair child development via direct effects on the central nervous system (CNS), they may also affect the developing brain via indirect pathways.^4^ For example, immune response to infection will lead to increased inflammatory changes while antibiotic treatment may affect the microbiome; both may be potential mechanisms negatively impacting neurodevelopment.^5 6^

Previous epidemiological studies have reported that childhood infections requiring hospitalisation are associated with an increased risk of adverse developmental and neurocognitive outcomes.^7^,^14^ However, these studies were limited by small or restricted cohorts, focused on specific infections, lack of socioeconomic status adjustment, variable outcome measures and incomplete assessment of school performance.^7^,^14^ Specifically, one study reporting an association was restricted to children born with extremely low birth weight^9^ while other studies did not take into account key confounding factors, including socioeconomic status and environmental factors.^11^,^13^

Given the limited evidence, we aimed to examine the association between infection requiring hospitalisation in early childhood and child development and school performance in a population-based study. We hypothesised that children with a history of hospitalisation for infection have an increased risk of developmental vulnerability and poor school performance compared with those not hospitalised for infection.

## Methods

### Study population and datasets

The study population included all singleton live births of ≥37 weeks gestation and ≥1500 g birth weight from 1 July 2001 to 31 December 2014 December, New South Wales (NSW), Australia, with a subsequent assessment of childhood development or school performance. NSW is the most populous state, comprising 31% of the national population. We only included term births as previous studies have shown that infants born preterm are more likely to have childhood infections and poor neurodevelopmental outcomes.^15^ Twins or multiple births and infants with major congenital conditions diagnosed in the first year of life^16^ or died before 9 years of age were also excluded. Given the two outcomes were ascertained using two unique data collections, linked data for children with each respective outcome were extracted to establish two separate cohorts (online supplemental appendix figure 1).

All births were identified from the Perinatal Data Collection (PDC), a legislated population-based surveillance system of all births in NSW (≥20 weeks gestation and ≥400 g birth weight). Information on childhood hospitalisations was ascertained from the NSW Admitted Patient Data Collection (APDC), a census of all public and private hospital admissions with a separate record for mothers at the time of birth, and for infants, information on every hospital admission from birth and during childhood. The APDC includes dates of admission, sociodemographic factors and up to 50 fields to record relevant diagnoses identified in each admission coded using the International Classification of Diseases version 10-Australian Modification (ICD10-AM).

The Australian Early Development Census (AEDC) is used to assess child development outcomes and school readiness based on the validated Early Development Instrument from Canada.^17^ The AEDC is a national assessment of child development for all children in their first year of full-time schooling (aged 4–6 years) conducted every 3 years since 2009. Teachers assess children on more than 100 items and scores for each are aggregated into five developmental domains: language and cognitive skill (school based), physical health and well-being, social competence, emotional maturity, and communication skills and general knowledge. Children with scores in the bottom 10th percentile of each domain are considered developmentally vulnerable, which was established in 2009.

School performance outcomes were obtained from the National Assessment Program-Literacy and Numeracy (NAPLAN), an annual nationwide assessment of all Australian children in grades 3, 5, 7 and 9. The first available NAPLAN results were used for this study with year 3 accounting for 98% of data. The two domains covering numeracy and reading were used because they provided the most stable results over time. The NAPLAN also records school factors and sociodemographic information such as parent education and occupation which were included in this study.^18^

Individual records for all births were probabilistically linked to the corresponding hospital, child development, and school performance datasets by the NSW Centre for Health Record Linkage. Data linkage is performed by the CheReL using ChoiceMaker software where information such as name, address and date of birth is matched across each dataset. These details are then replaced with a unique person project number (PPN) and provided to the researcher with content data for each dataset. The PPN is then used to merge records across datasets. The linkage completed by CheReL resulted in a false positive rate of 0.5%.

### Study outcomes, exposure and confounders

Our study identified two key outcomes: (1) those children defined by the AEDC as developmentally high risk (DHR), classified as developmentally vulnerable in at least two of the five development domains assessed^18^ and (2) those with NAPLAN results for reading or numeracy in the lowest of six bands, classified as children performing below the national minimum standard.^19 20^ Developmental vulnerability in each of the five domains from AEDC was also assessed.

Our primary exposure to interest was infection-related hospitalisation. This was obtained from the APDC and based on definitions and ICD10-AM coding established in previous studies (online supplemental appendix table 1).^21 22^ We only included infections recorded as the primary diagnosis of patients admitted to the hospital during the first 4 years of life. Infection-related hospitalisation was also grouped by type of infection (upper respiratory tract infection, lower respiratory tract infection, infectious enteritis, skin infection, urinary tract infection, CNS for organ-specific infection, sepsis, vaccine-preventable infections and infection requiring intensive care unit (ICU) admission). For sepsis, all diagnosis fields were used as not all sepsis may be coded as the primary reason for hospital admission. We also assessed infection-related hospitalisations by several criteria and categorised these by age of first admission (<1, 1–2, 2–3 and 3–4 years), length of stay of first infection-related hospitalisation (≤1, 2 and 3+ days) and the number of infection-related hospitalisations (1, 2 and 3+). Hospital admissions for infection that occurred within 14 days of a previous infection-related hospitalisation were considered as a single infection admission. Unexposed children were defined as those not hospitalised for infection in the first 4 years of life.

Potential confounding variables included maternal characteristics (maternal age at birth, smoking during pregnancy, maternal diabetes, maternal country of birth, maternal remoteness and quintile of socioeconomic disadvantage (based on Socio-Economic Index for Areas, SEIFA), and mode of childbirth from the PDC; maternal education and occupation from NAPLAN datasets), paternal characteristics (education and occupation from NAPLAN data), and birth and child characteristics (sex, birth weight, gestational age, year of birth, season of birth from PDC and severe neonatal morbidity, paediatric comorbidity and cumulative length of hospital stay from APDC). Severe neonatal comorbidity was defined as a wide range of conditions and procedures observed in severely ill infants at birth or in the first 28 days of life.^23^ Paediatric comorbidity was defined as children hospitalised for any chronic conditions recorded in the primary diagnosis field from 28 days of birth to 9 years of age according to Hardelid *et al *.^24^ Cumulative length of hospital stay was counted as total hospital length of stay in the first 4 years of life excluding the birth admission and categorised into four groups (0, 1–3, 4–7 and 8+ days).

### Statistical analysis

As children have different lengths of exposure periods between infection and outcome, the association between infection-related hospitalisation and outcome was examined using a time-varying Cox proportional hazards regression model to estimate HRs. The follow-up time started from the date of birth and ended at the date of early child development assessment or the first record of school performance in each of the two cohorts. At the start of follow-up, all children were initially classified as having no infection-related hospitalisation and then contributed person-time to the categories of exposure group from the date of the first record of infection-related hospitalisation. All HRs were adjusted for maternal, paternal, and birth and child characteristics and we used a robust sandwich variance estimator to account for clustering of children within the same school. Additional analyses for various exposures were conducted by type of infection, age at first infection, length of hospital stay for first infection-related hospitalisation and the number of infection-related hospitalisations.

A sensitivity analysis was conducted for the primary outcome by including infection-related hospitalisation from all fields of diagnosis. Potential unmeasured confounding was assessed by calculating E-values (EValue R package).^25^

Sibling analysis was conducted by restricting to children with siblings who were born to the same mother to examine the role of confounding by shared home environment and genetic factors using conditional logistic regression, clustering on maternal identifier as the family indicator. The sibling analysis compared the outcomes among children with exposure to infection-related hospitalisation and the outcomes among their siblings closest in age without exposure to infection-related hospitalisation using fixed effects models. We also conducted stratified analysis by type, age, number and length of hospital stay of infection.

Data were analysed by using SAS V.9.4 and R V.4.1.3 with estimates reported using HR (or ORs) and 95% CIs with statistical significance indicated when the null (one) was not included in the range.

## Results

A total of 276 454 children were included in the cohort of child development outcome, and 644 291 infants in the cohort with NAPLAN school performance outcome (table 1). Median follow-up for developmental outcome was 5 years (IQR 5–6) and for school performance was 9 years (IQR 8–9). For both cohorts, approximately 21% (n=516 110 and 135 095, respectively) of children had at least one hospital admission for infectious disease as the principal diagnosis (table 1). Compared with children without an infection-related hospitalisation, children were more likely to be hospitalised for infection for the following groups: those born to younger mothers, mothers with lower education levels, mothers residing in areas with lower socioeconomic disadvantage, children being male, those with severe neonatal morbidity and those with paediatric comorbidity (table 1).

**Table 1:** Maternal, birth and child characteristics for child development and school performance by infection requiring hospitalisation

	Cohort for development (N=276 454)		Cohort for education (N=644 291)	
	Not-hospitalised for infection (%)	Hospitalised for infection (%)	Not-hospitalised for infection (%)	Hospitalised for infection (%)
Overall	220 344 (79.7)	56 110 (20.3)	509 196 (79.0)	135 095 (21.0)
Maternal age at birth (years)				
≤19	6893 (3.1)	2545 (4.5)	16 573 (3.3)	6531 (4.8)
20–24	29 018 (13.2)	8994 (16.0)	68 302 (13.4)	22 268 (16.5)
25–29	60 218 (27.3)	15 732 (28.0)	139 787 (27.5)	38 141 (28.2)
30–34	74 014 (33.6)	17 837 (31.8)	170 812 (33.5)	42 520 (31.5)
35–39	41 303 (18.7)	9109 (16.2)	94 196 (18.5)	21 412 (15.8)
40+	8898 (4.0)	1893 (3.4)	19 526 (3.8)	4223 (3.1)
Maternal smoking during pregnancy				
No	195 325 (88.6)	47 964 (85.5)	447 063 (87.8)	114 012 (84.4)
Yes	25 019 (11.4)	8146 (14.5)	62 133 (12.2)	21 083 (15.6)
Gestational or maternal diabetes				
No	207 276 (94.1)	52 967 (94.4)	483 084 (94.9)	128 434 (95.1)
Yes	13 068 (5.9)	3143 (5.6)	26 112 (5.1)	6661 (4.9)
Maternal country of birth				
Australia	153 388 (69.6)	41 807 (74.5)	360 342 (70.8)	102 314 (75.7)
Overseas	66 956 (30.4)	14 303 (25.5)	148 854 (29.2)	32 781 (24.3)
Maternal residential location				
Major city	171 328 (77.8)	42 368 (75.5)	398 572 (78.3)	101 998 (75.5)
Inner regional	37 355 (17.0)	10 249 (18.3)	84 403 (16.6)	24 613 (18.2)
Outer regional or more	11 661 (5.3)	3493 (6.2)	26 221 (5.1)	8484 (6.3)
SEIFA				
Q1 (most disadvantage)	40 675 (18.5)	11 521 (20.5)	95 935 (18.8)	28 403 (21.0)
Q2	43 780 (19.9)	12 030 (21.4)	101 040 (19.8)	28 877 (21.4)
Q3	48 249 (21.9)	11 496 (20.5)	110 410 (21.7)	27 316 (20.2)
Q4	40 786 (18.5)	9574 (17.1)	94 886 (18.6)	23 029 (17.0)
Q5	46 854 (21.3)	11 489 (20.5)	106 925 (21.0)	27 470 (20.3)
Maternal education				
University	46 877 (21.3)	10 889 (19.4)	155 263 (30.5)	35 787 (26.5)
Certificate	59 167 (26.9)	16 102 (28.7)	196 651 (38.6)	53 865 (39.9)
Year 12	14 612 (6.6)	3777 (6.7)	49 318 (9.7)	12 735 (9.4)
Below year 12	21 016 (9.5)	6427 (11.5)	71 428 (14.0)	21 802 (16.1)
Unknown/not stated	78 672 (35.7)	18 915 (33.7)	36 536 (7.2)	10 906 (8.1)
Paternal education				
University	38 126 (17.3)	8512 (15.2)	132 490 (26.0)	29 372 (21.7)
Certificate	58 751 (26.7)	15 511 (27.6)	195 732 (38.4)	51 935 (38.4)
Year 12	10 936 (5.0)	2786 (5.0)	37 748 (7.4)	9576 (7.1)
Below year 12	16 205 (7.4)	4924 (8.8)	55 813 (11.0)	16 736 (12.4)
Unknown/not stated	96 326 (43.7)	24 377 (43.4)	87 413 (17.2)	27 476 (20.3)
Maternal occupation				
Managers/professionals	50 840 (23.1)	12 832 (22.9)	170 300 (33.4)	43 143 (31.9)
Tradesperson	29 130 (13.2)	7792 (13.9)	97 099 (19.1)	26 084 (19.3)
Other paid workers	16 275 (7.4)	4523 (8.1)	56 200 (11.0)	15 127 (11.2)
Non-paid workers	38 567 (17.5)	10 066 (17.9)	123 608 (24.3)	32 180 (23.8)
Unknown/not stated	85 532 (38.8)	20 897 (37.2)	61 989 (12.2)	18 561 (13.7)
Paternal occupation				
Managers/professionals	60 107 (27.3)	14 690 (26.2)	203 370 (39.9)	49 196 (36.4)
Tradesperson	30 318 (13.8)	7729 (13.8)	100 757 (19.8)	26 120 (19.3)
Other paid workers	23 648 (10.7)	6555 (11.7)	77 121 (15.1)	21 157 (15.7)
Non-paid workers	5762 (2.6)	1613 (2.9)	25 715 (5.1)	6719 (5.0)
Unknown/not stated	100 509 (45.6)	25 523 (45.5)	102 233 (20.1)	31 903 (23.6)
Sex				
Male	107 005 (48.6)	30 679 (54.7)	248 275 (48.8)	73 927 (54.7)
Female	113 339 (51.4)	25 431 (45.3)	260 921 (51.2)	61 168 (45.3)
Gestational age, week				
37–38	51 759 (23.5)	14 673 (26.2)	114 547 (22.5)	34 033 (25.2)
39–41	165 676 (75.2)	40 743 (72.6)	386 342 (75.9)	99 112 (73.4)
42+	2909 (1.3)	694 (1.2)	8307 (1.6)	1950 (1.4)
Birth weight (g)				
1500–2499	3365 (1.5)	922 (1.6)	7858 (1.5)	2351 (1.7)
2500–3499	114 668 (52.0)	28 604 (51.0)	262 408 (51.5)	68 854 (51.0)
3500–4499	98 282 (44.6)	25 481 (45.4)	229 337 (45.0)	61 226 (45.3)
4500+	4029 (1.8)	1103 (2.0)	9593 (1.9)	2664 (2.0)
Mode of delivery				
Vaginal	159 184 (72.2)	39 431 (70.3)	374 308 (73.5)	96 687 (71.6)
Caesarean section	61 061 (27.7)	16 660 (29.7)	134 688 (26.5)	38 353 (28.4)
Cumulative length of hospital stay up to 4 years, days				
0	180 472 (81.9)	3519 (6.3)	414 184 (81.3)	8397 (6.2)
1–3	28 531 (12.9)	28 340 (50.5)	68 062 (13.4)	67 415 (49.9)
4–7	9153 (4.2)	16 604 (29.6)	21 369 (4.2)	40 202 (29.8)
8+	2188 (1.0)	7647 (13.6)	5581 (1.1)	19 081 (14.1)
Severe neonatal morbidity				
No	216 954 (98.5)	54 348 (96.9)	502 028 (98.6)	131 036 (97.0)
Yes	3390 (1.5)	1762 (3.1)	7168 (1.4)	4059 (3.0)
Comorbidity in childhood				
No	210 618 (95.6)	49 867 (88.9)	483 095 (94.9)	118 199 (87.5)
Yes	9726 (4.4)	6243 (11.1)	26 101 (5.1)	16 896 (12.5)
Year of birth*				
2001–2007	103 993 (47.2)	28 329 (50.5)	270 873 (53.2)	77 057 (57.0)
2008–2014	116 351 (52.8)	27 781 (49.5)	238 323 (46.8)	58 038 (43.0)
Season of birth				
Spring	56 318 (25.6)	13 770 (24.5)	132 751 (26.1)	33 880 (25.1)
Summer	52 844 (24.0)	13 551 (24.2)	124 059 (24.4)	32 906 (24.4)
Autumn	55 325 (25.1)	14 709 (26.2)	123 805 (24.3)	34 596 (25.6)
Winter	55 857 (25.3)	14 080 (25.1)	128 581 (25.3)	33 713 (25.0)

* For school performance, year of birth werewas categorizedcategorised into 2001–2006 and 2007–2011.

SEIFA, Socio-Economic Index for Areas

For child development outcomes, compared with children without an infection-related hospitalisation, a higher proportion of children with infection-related hospitalisation were developmentally vulnerable in each of the five domains of child development and were DHR (10.9% vs 8.7%) (table 2). For school performance outcome, a higher proportion of children with hospitalisation for infection had numeracy (3.7% vs 2.7%) and reading results (4.3% vs 3.1%) below the national standard. After adjusting for maternal and birth and child characteristics, children with infection-related hospitalisation were more likely to be developmentally vulnerable in all five domains of developmental outcomes (adjusted HR, aHR in the range of 1.07 and 1.16), DHR (aHR 1.12, 95% CI 1.09 to 1.16) and at increased risk of having scores below the national minimum standard for numeracy (aHR 1.25, 95% CI 1.21 to 1.30) and reading (aHR 1.19, 95% CI 1.15 to 1.22) (table 2). These results were similar following additional adjustment for socioeconomic factors (table 2). Consistent with infection-related hospitalisation identified from primary diagnosis of hospital admission, similar associations were found when infections were identified from all fields of hospital admissions (online supplemental appendix table 2).

**Table 2:** Association of childhood infection requiring hospitalisation with child development and school performance

Child development and school performance outcomes	Cases (% among unexposed)	Cases (% among exposed)	HR (95% CI)	Adjusted HR* (95% CI)	Adjusted HR† (95% CI)
Child development (n=276 454)					
Age at assessment, mean (SD), years	5.2 (0.4)	5.2 (0.4)			
Developmentally high risk	19 162 (8.7)	6113 (10.9)	1.22 (1.18 to 1.25)	1.12 (1.09 to 1.16)	1.12 (1.08 to 1.15)
Developmental vulnerability in the five domains					
Language and cognitive skills (school based)	10 097 (4.6)	3360 (6.0)	1.27 (1.21 to 1.32)	1.16 (1.11 to 1.21)	1.15 (1.10 to 1.20)
Physical health and well-being	17 040 (7.7)	5347 (9.5)	1.20 (1.16 to 1.24)	1.12 (1.09 to 1.16)	1.12 (1.08 to 1.15)
Social competence	17 943 (8.1)	5683 (10.1)	1.21 (1.17 to 1.24)	1.13 (1.09 to 1.16)	1.12 (1.09 to 1.16)
Emotional maturity	14 044 (6.4)	4468 (8.0)	1.21 (1.17 to 1.25)	1.10 (1.06 to 1.14)	1.09 (1.05 to 1.13)
Communication skills and general knowledge	15 930 (7.2)	4617 (8.2)	1.11 (1.07 to 1.15)	1.07 (1.03 to 1.10)	1.06 (1.03 to 1.10)
School performance (n=644 291)					
Age at test, mean (SD), years	8.1 (0.6)	8.1 (0.6)			
Numeracy below national minimum standard	13 452 (2.7)	5024 (3.7)	1.37 (1.32 to 1.42)	1.25 (1.21 to 1.30)	1.22 (1.18 to 1.26)
Reading below national minimum standard	15 873 (3.1)	5774 (4.3)	1.33 (1.29 to 1.38)	1.19 (1.15 to 1.22)	1.16 (1.12 to 1.20)

* Adjusted for maternal age, maternal smoking, maternal diabetes, maternal country of birth, maternal residence, sex of infant, gestational age, birth weight, delivery mode, severe neonatal morbidity, year of birth, season of birth.

† Additionally adjusted for Socio-Economic Index for Areas (), maternal education and occupation, paternal education, and occupation.

SDstandard deviation

The E-value for unmeasured confounding for infection-related hospitalisation was 1.48 (a lower 95% CI 1.37) for DHR, 1.74 (a lower 95% CI 1.64) for numeracy, 1.59 (a lower 95% CI 1.49) for reading. We also found the risk estimates for the association between some covariates and primary outcomes were higher than those for infection, including maternal age <25 years, maternal smoking and male sex for DHR, and mothers residing in an area with low socioeconomic status (Q1, Q2), maternal and paternal education lower than tertiary level and maternal and parental unpaid occupations (online supplemental appendix table 3).

**Figure 1:**
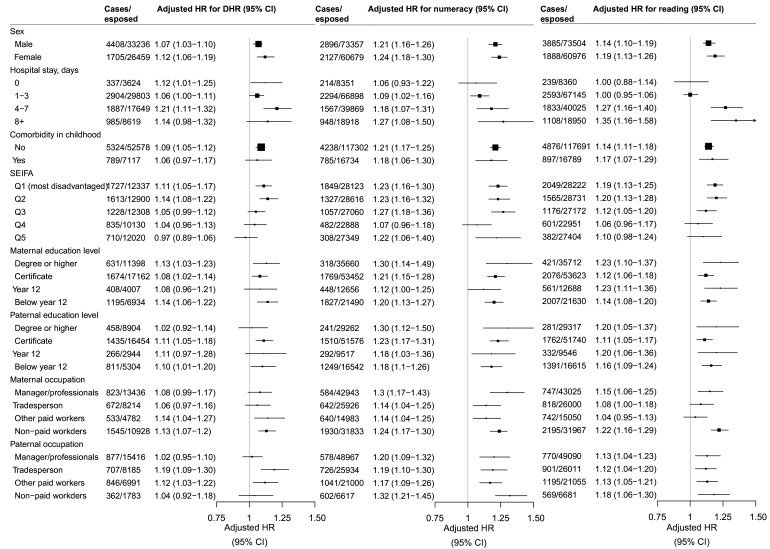
Stratified analysis for development and school performance among children with infection-related hospitalisation compared with children without infection-related hospitalisation. Adjusted for maternal age, maternal smoking, maternal diabetes, maternal country of birth, maternal residence, maternal Socio-Economic Index for Areas (SEIFA), maternal education and occupation, paternal education and occupation, sex of infant, gestational age, birth weight, delivery mode, severe neonatal morbidity, year of birth, season of birth. CNS, central nervous system; DHR, developmentally high-risk.

In the stratified analysis (figure 1), we did not find the associations that differed by sex or comorbidity in childhood for any of our outcomes. For cumulative length of hospital stay in the first 4 years of life, there was no difference for DHR, but stronger associations for numeracy and reading in those children with longer hospital stay 4–7 and 8+ days. While significant associations for the three major outcomes were consistently found in the lowest two quintiles of SEIFA (the more disadvantaged groups), no consistent association was found in the more advantaged groups.

**Figure 2:**
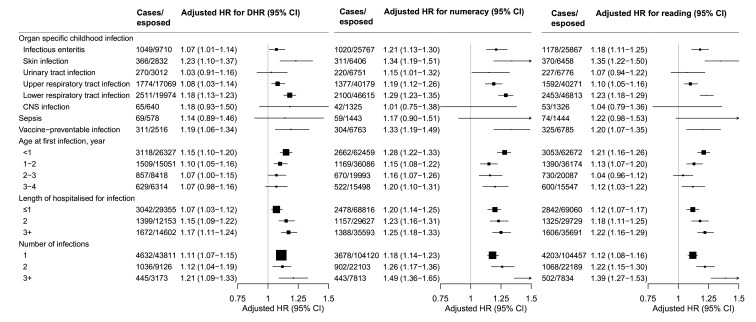
Associations of infection-related hospitalisation with child development and school performance by different categories of infection. Adjusted for maternal age, maternal smoking, maternal diabetes, maternal country of birth, maternal residence, Socio-Economic Index for Areas (SEIFA), maternal education and occupation, paternal education and occupation, sex of infant, gestational age, birth weight, delivery mode, severe neonatal morbidity, year of birth, season of birth. Due to the small number of ICU admission, the results were not presented in the figure but described in the manuscript. CNS, central nervous system; DHR, developmentally high risk; ICU, intensive care unit.

In the analyses by type of infection (figure 2), increased risk of developmental vulnerability and poor school performance was found for skin infection (aHR 1.23–1.35), lower respiratory tract infection (aHR 1.18–1.29) and vaccine-preventable infections (aHR 1.19–1.33). No association was found for other types of infection. Analysis by age and length of hospital stay for first infection-related hospitalisation showed the highest risk for children infected in their first year of life and with longer hospital stay. The association was found stronger with an increasing number of infections for DHR (aHR 1.11–1.21), numeracy (aHR 1.18–1.49) and reading (aHR 1.12–1.39).

### Sibling cohort

The discordant-matched siblings comprised 18 006 and 109 754 sibling pairs requiring hospitalisation for the child development and school performance cohorts, respectively (online supplemental appendix figure 1). The distribution of characteristics from the sibling cohort was similar to the population-based cohort for child development and school performance (online supplemental appendix tables 4,5). Crude univariable analysis showed a small increased association between childhood infection and study outcomes. Following multivariable analysis, these results were attenuated to null except for a modest association with numeracy outcome (aOR 1.15; 95% CI 1.07 to 1.23) (table 3). For both cohorts, male sex was the only factor consistently associated with all study outcomes (aOR 3.11, 95% CI 2.59 to 3.74 for DHR; aOR 1.20, 95% CI 1.08 to 1.33 for numeracy; aOR 2.09, 95% CI 1.88 to 2.31 for reading) (online supplemental appendix table 6). In the stratified analysis, significant associations were found for children born to mothers from the lowest two quintiles of SEIFA for DHR and numeracy outcome and being male for numeracy outcome (online supplemental appendix table 7). In the analysis by different categories of infection, we found type of infection (lower respiratory tract infection), number of infections and age of infection were associated with modest effects on outcomes, but the estimates might be imprecise due to small numbers (online supplemental appendix table 8).

**Table 3:** Association of infection-related hospitalisation with child development and school performance in sibling cohort

	Cases (% among unexposed)	Cases (% among exposed)	OR (95% CI)	Adjusted OR* (95% CI)
Child Development (n=18 006)				
Developmentally high risk	862 (9.6)	941 (10.5)	1.12 (1.01 to 1.25)	1.03 (0.91 to 1.16)
Developmental vulnerability in the five domains				
Language and cognitive skills (school based)	520 (5.8)	557 (6.2)	1.09 (0.95 to 1.25)	1.04 (0.89 to 1.22)
Physical health and well-being	797 (8.9)	802 (8.9)	1.01 (0.90 to 1.13)	0.95 (0.84 to 1.07)
Social competence	780 (8.7)	860 (9.6)	1.13 (1.01 to 1.25)	1.04 (0.92 to 1.17)
Emotional maturity	554 (6.2)	633 (7.0)	1.16 (1.03 to 1.32)	1.06 (0.91 to 1.22)
Communication skills and general knowledge	710 (7.9)	771 (8.6)	1.11 (0.99 to 1.25)	1.07 (0.94 to 1.21)
School performance (n=109 754)				
Numeracy below national minimum standard	1697 (3.1)	1932 (3.5)	1.16 (1.08 to 1.25)	1.15 (1.07 to 1.24)
Reading below national minimum standard	2121 (3.9)	2247 (4.1)	1.07 (1.01 to 1.14)	1.01 (0.95 to 1.08)

* Adjusted for maternal age, maternal smoking, maternal diabetes, maternal country of birth, maternal residence, Socio-Economic Index for Areas (), maternal education and occupation, paternal education and occupation, sex of infant, gestational age, birth weight, delivery mode, severe neonatal morbidity, year of birth, season of birth, and birth order. OR: odds ratio

ORodds ratio

## Discussion

Childhood infection requiring hospitalisation was found to have a modest association with child developmental vulnerability and poor school performance. Associations were stronger for specific types of infection (skin, lower respiratory tract and vaccine-preventable infections) and for children with three or more infections. However, results may be overestimated due to unmeasured confounding and no significant associations following sibling cohort analysis. These findings were potentially explained by shared familial factors, particularly in families with the most socioeconomic disadvantages.

Our findings in the two population-based cohorts are consistent with previous studies,^9^,^12^ suggesting that children with hospitalised infection had increased risk of poor development and school performance. However, such associations were not found in the sibling analysis in this study, which was not conducted by previous studies. Findings highlight that shared familial or environmental factors in the home have a stronger impact than childhood infection on poor child development and school outcomes. However, some experts note that estimates from a sibling analysis may be biased by non-shared confounders if siblings are less similar with regard to confounders than to the exposure.^26^ In addition, random measurement error in exposure might also be higher in the sibling analysis, thus leading the associations to be attenuated.^27^ However, the exposure to infection-related hospitalisation in our study is less likely to be subject to measurement error due to health-seeking behaviour as hospital care is universal and free in Australia. It is important that future studies also apply a sibling design to determine the role of infection after taking into account family-level effects.

The overestimated impact of childhood infection on development outcome and school performance was further supported by our assessment of unmeasured confounding in the full cohort using E-values. We found that an unmeasured confounder would need an association of at least 1.37 to 1.64 (lower 95% CI) with both infection-related hospitalisation and DHR, or poor reading or numeracy scores in order to explain away the observed association. Furthermore, we found some of the sociodemographic factors in our models had risk estimates of up to 2.5, including residence in most disadvantaged areas, maternal and paternal high school education and fathers in non-paid work, highlighting the importance of socioeconomic factors in explaining poor child development and school performance. Previous studies have also shown that sociodemographic factors have the strongest effect on school outcomes and a focus on addressing underlying social disadvantage is required to have an impact on child outcomes.^28 29^

Our findings are also novel in that we investigated and highlighted the association of specific types of infections and increased number of infections, as a marker of severity, in a child associated with greater developmental vulnerability and poor school performance. Poorer outcomes were more likely for skin, infectious enteritis, lower respiratory tract and vaccine-preventable infections. Several biological mechanisms have been proposed and studied in animal or clinical studies. The first one is the direct impairment of brain development. Studies have suggested some infections including CNS infection could result in functional disorders including neurodevelopment disorder, gross-motor function delay and intellectual disability.^30 31^ While no significant association was found in our study, this may be due to our small sample sizes for CNS infections, suggesting a study with a larger sample size is needed in the future. In addition, some infections (infectious enteritis) and repeated antibiotic exposures to treat infections might disrupt the composition of gut microbiota to adversely affect neurodevelopment in children as suggested by a recent study.^32^ This is supported by the findings that enteric pathogens detected from stool samples were negatively related to child cognitive development in low-resource settings.^33^ A study from Brazil indicated that early childhood diarrhoea may have independent effects on children’s intellectual function in later childhood.^34^ However, the mechanism of how these infections adversely affect children’s neurodevelopment remains unknown. It is, therefore, important to identify pathogen-specific infection and their relation to developmental outcomes in future studies.

Our study also adds to knowledge about the potential negative impact of some vaccine-preventable infections in early life on child development and school performance. Previous studies have suggested that prenatal influenza exposure and neonatal rotavirus infection were associated with an increased risk of poorer cognitive performance.^35 36^ In this study, rotavirus infection and influenza accounted for almost three-quarters (73%) of all vaccine-preventable infections (online supplemental appendix table 9). While vaccine data were not available, it would be interesting to see whether receipt of a vaccine against influenza or rotavirus is associated with a lower risk of poor developmental outcomes compared with children without receipt of these vaccines.

The main strength of this study is the population based, and large cohort with full birth records and long follow-up into childhood. Our findings were strengthened by the adjustment of socioeconomic and familial factors. Our study also investigated school performance and demonstrated consistency and robustness of findings to those for child development in an independent outcome assessed 3–4 years later. Consistent findings were further supported by a range of different stratified analyses in the main and sibling cohorts. One of the key limitations of this study is the lack of detailed information about specific pathogens causing these infection-related hospitalisations. In addition, only infections admitted to the hospital were included in this study and do not account for those with milder infections not requiring hospitalisation. Therefore, those included in our study represent the more severely affected and potentially at risk of adverse long-term outcomes. Another limitation is that we did not have information about school absenteeism. In addition, unmeasured confounding might still exist in our analysis although we adjusted for a range of potential confounders identified from our comprehensive linked records. This may be supported by the attenuated observed associations in the sibling cohort analyses, suggesting shared but unmeasured environmental or genetic factors potentially mediate the association.

In conclusion, despite the modest effect of childhood infection, given it is more likely to occur in families with more socioeconomic disadvantage, focusing on these families at risk to prevent and reduce childhood infection and, more broadly, social disadvantage may help improve long-term child development and school performance.

## supplementary material

10.1136/jech-2024-222040 online supplemental file 1

## Data Availability

Data may be obtained from a third party and are not publicly available.
