# Supplementary material for: Impact of early childhood infection on child development and school performance: a population-based study
Source: J Epidemiol Community Health. 2024 Aug 31;79(1):e222040. doi: 10.1136/jech-2024-222040 (PMC11671983; doi:10.1136/jech-2024-222040)
Supplement: online supplemental file 1 [file jech-79-1-s001.pdf]

Appendix Figure 1. Flowchart of study population for main and sibling analysis of early child development and school performance

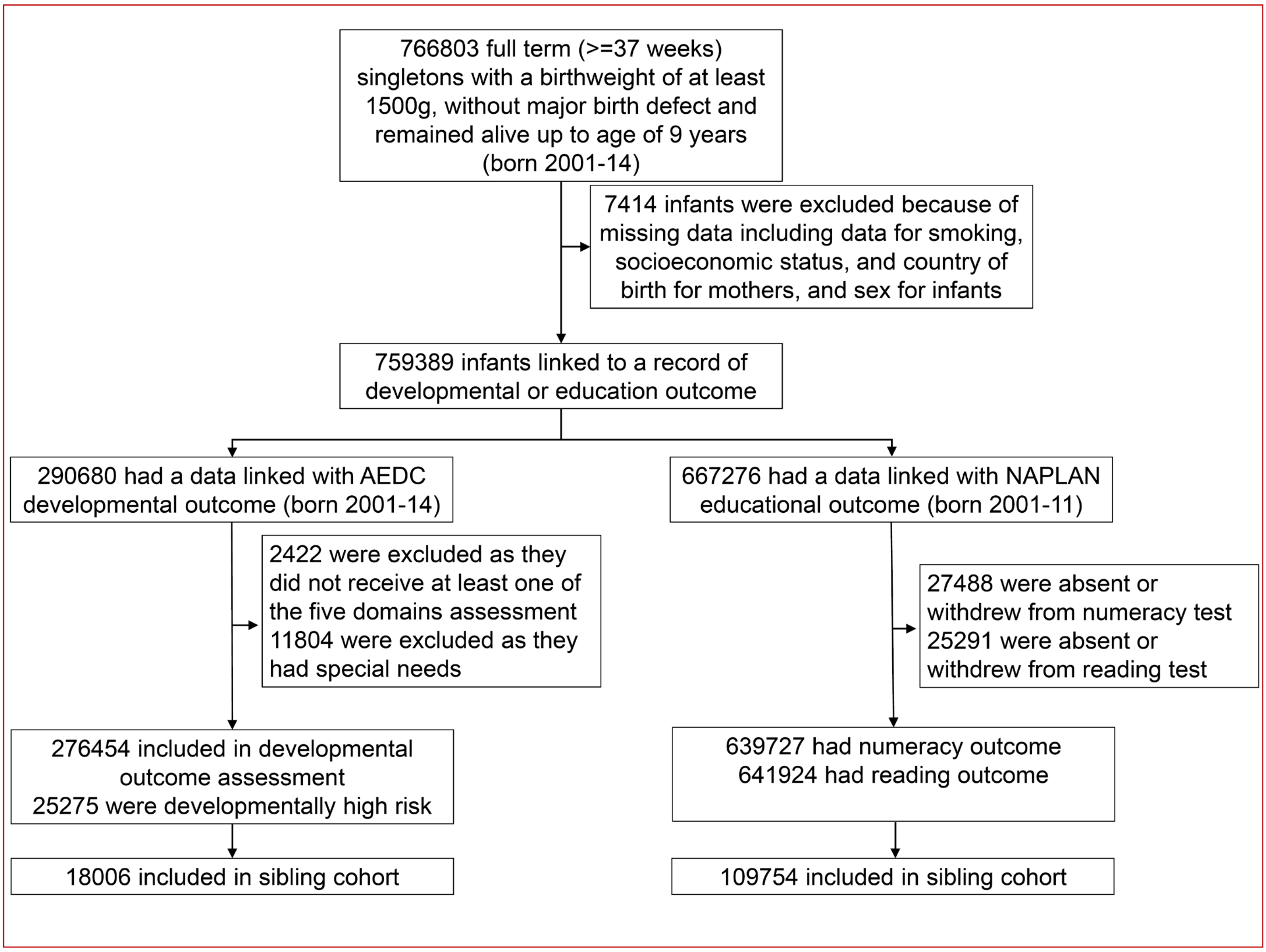

AEDC=Australian Early Development Census. NAPLAN=National Assessment Program Literacy and Numeracy.

Appendix Table 1. ICD10-AM diagnosis codes of infectious disease categories, modified from Miller et al 2020.

Type of infection	ICD10-AM code
**All infections:**	A0, A1, A2, A3, A4, A5, A6, A7, A9, B0, B1, B2, B3, B4, B5, B6, B7, B8, J0, L0, A81, A82, A83, A84, A85, A86, A87, A89, B95, B96, B97, B98, B99, G01, G02, G03, G04, G05, G06, G07, G08, G92, H00, H65, H66, H70, H72, I00, I01, I39, J11, J12, J13, J14, J15, J16, J17, J20, J21, J22, J32, J36, J40, J41, J42, J47, J65, J85, K61, K65, K67, L88, M00, M01, N33, N45, N51, N61, N73, N74, O23, O85, O86, O91, O98, P23, P35, P36, P37, P38, P39, Z21, A801, A802, A803, A804, A809, A880, A888, D593, G940, H010, H018, H030, H031, H040, H061, H100, H102, H103, H105, H130, H131, H190, H191, H192, H200, H220, H320, H440, H441, H451, H481, H588, H600, H601, H602, H603, H620, H621, H622, H623, H670, H671, H680, H730, H750, H830, H921, H940, I301, I320, I321, I330, I400, I410, I411, I412, I430, I520, I521, I681, I980, I981, J180, J181, J188, J189, J340, J350, J390, J391, J392, J440, J998, K044, K045, K046, K047, K052, K053, K054, K113, K122, K528, K630, K750, K751, K770, K810, K871, K908, K930, K931, L130, L303, L540, M462, M463, M464, M465, M490, M491, M492, M493, M600, M630, M631, M632, M650, M651, M680, M710, M711, M726, M728, M730, M731, M860, M861, M862, M864, M865, M868, M869, M900, M901, N080, N088, N136, N151, N159, N160, N220, N291, N300, N308, N340, N351, N390, N410, N412, N431, N700, N750, N751, N760, N764, N766, N768, N770, N771, O030, O035, O040, O045, O050, O055, O060, O065, O070, O075, O080, O264, O411, O753, P002, P027, M6009, M8660, M8667, M8668, M8669
**Vaccine-preventable infection**	J10, J11, J13, J14, A080, A35, A36, A37, A80, B01, B05, B06, B161, B169, B180, B181, B26, G000, J09, A33, A34, B170, B05, B189, B19
**Infectious enteritis**	A00, A01, A03, A04, A05, A07, A08, A09, A020, A022, A028, A029, A060, A061, A062, A063, A064, A069, A213, A222, B710, B719, B760, B789, B810, K528, B3788, B9681
**Skin infection**	A46, B35, B85, B86, B88, L00, L02, L03, L05, L08, L88, B000, B360, B361, B362, B363, B368, B372, B879, H603, L010, L130, L303, M630, M631, M632, M650, M651, M680, M710, M711, P390, P394, M6009, O9100, O9110
**Urinary tract infection**	A51, A53, A54, A55, A56, A57, A58, A60, A63, A64, N45, A368, A529, A590, B373, B374, N136, N151, N300, N301, N302, N308, N340, N351, N390, N410, N412, N431, N512, N700, N730, N731, N732, N733, N734, N739, N742, N743, N744, N750, N751, N760, N764, O030, O035, O080, O264, O862, O981, O982, P393
**Upper respiratory tract infection**	H65, H66, H70, H72, J00, J01, J02, J03, J04, J06, J32, J36, A360, A361, A362, B053, H670, H678, H680, H730, H750, H830, H921, J051, J340, J350
**Lower respiratory tract infection**	A15, A16, A37, A70, B39, B59, J09, J12, J13, J14, J15, J16, J17, J20, J21, J22, J40, J41, J42, J47, J65, J86, A192, A198, A199, A202, A212, A221, A240, A310, A420, A481, B012, B052, B340, B371, B380, B381, B382, B400, B401, B402, B403, B407, B408, B410, B420, B440, B441, B450, B583, J050, J100, J101, J110, J111, J180, J181, J188, J189, J440, J850, J851, J852, P230, P232, P233, P234, P235, P236, P238, P239
**Central nervous system infection**	A17, A321, A39, A8, B003, B004, B010, B011, B020, B021, B050, B051, B060, B261, B262, B375, B582
**Sepsis**	A40, A41

Appendix Table 2. Association of hospitalization for infection from any field of diagnosis with child development and school performance outcomes.

	Case/exposed	HR (95% CI)	Adjusted HR (95% CI)
Development (n=276454)			
Development high-risk	6955/64240	1.22 (1.18-1.25)	1.12 (1.08-1.15)
Developmental vulnerability in the five domains			
Language and cognitive skills (school based)	3804/64240	1.26 (1.21-1.31)	1.15 (1.10-1.19)
Physical health and wellbeing	6107/64240	1.21 (1.17-1.24)	1.12 (1.09-1.16)
Social competence	6458/64240	1.21 (1.17-1.24)	1.12 (1.08-1.15)
Emotional maturity	5070/64240	1.20 (1.16-1.25)	1.08 (1.05-1.12)
Communication skills and general knowledge	5272/64240	1.11 (1.08-1.15)	1.06 (1.03-1.10)
School performance (n=644291)			
Numeracy lower than national standard	5579/152730	1.34 (1.29-1.39)	1.21 (1.17-1.25)
Reading lower than national standard	6478/153249	1.32 (1.28-1.37)	1.16 (1.12-1.19)

Adjusted for maternal age, maternal smoking, maternal diabetes, maternal country of birth, maternal residence, Socio-Economic Index for Areas (SEIFA), maternal education and occupation, paternal education and occupation, sex of infant, gestational age, birth weight, delivery mode, severe neonatal morbidity, year of birth, season of birth.

Appendix Table 3. Associations of maternal, infant and birth characteristics with child development and school performance.

Maternal, infant and birth characteristics	Adjusted HR for DHR (95%CI)	Adjusted HR for numeracy (95%CI)	Adjusted HR for reading (95%CI)
Infectious disease	1.12 (1.08-1.15)	1.22 (1.18-1.26)	1.16 (1.12-1.20)
Maternal age, years			
<=19	1.89 (1.78-2.00)	1.35 (1.27-1.44)	1.35 (1.27-1.43)
20-24	1.57 (1.51-1.63)	1.32 (1.26-1.38)	1.28 (1.23-1.34)
25-29	1.14 (1.10-1.18)	1.13 (1.08-1.18)	1.15 (1.11-1.19)
30-34	1 (Reference)	1 (Reference)	1 (Reference)
35-39	1.03 (0.99-1.07)	0.98 (0.93-1.03)	0.96 (0.92-1.01)
40+	1.15 (1.08-1.24)	0.98 (0.89-1.08)	0.95 (0.87-1.04)
Smoking during pregnancy	1.59 (1.53-1.65)	1.38 (1.32-1.43)	1.39 (1.34-1.45)
Maternal diabetes	1.16 (1.10-1.22)	1.16 (1.09-1.24)	1.15 (1.08-1.22)
Mothers born overseas	1.69 (1.62-1.75)	1.21 (1.15-1.28)	1.03 (0.98-1.09)
Residence			
Major city	1 (Reference)	1 (Reference)	1 (Reference)
Inner regional	0.80 (0.76-0.84)	0.78 (0.72-0.83)	0.92 (0.87-0.98)
Outer regional or remote	0.82 (0.75-0.89)	0.92 (0.83-1.01)	1.05 (0.96-1.14)
SEIFA			
Q1 (most disadvantaged)	1.77 (1.64-1.91)	2.62 (2.36-2.91)	2.21 (2.01-2.42)
Q2	1.56 (1.45-1.67)	2.05 (1.86-2.26)	1.81 (1.66-1.98)
Q3	1.37 (1.28-1.47)	1.90 (1.73-2.10)	1.70 (1.56-1.86)
Q4	1.29 (1.20-1.38)	1.55 (1.41-1.71)	1.47 (1.35-1.61)
Q5	1 (Reference)	1 (Reference)	1 (Reference)
Male sex	1.93 (1.87-1.98)	1.01 (0.97-1.04)	1.54 (1.50-1.59)
Gestational age, week			
37-38	1.07 (1.04-1.10)	1.02 (0.98-1.06)	0.97 (0.93-1.00)
39-41	1 (Reference)	1 (Reference)	1 (Reference)
42+	1.00 (0.89-1.12)	1.01 (0.90-1.14)	0.96 (0.86-1.08)
Birthweight, gram			
1500-2499	1.18 (1.09-1.29)	1.26 (1.16-1.37)	1.21 (1.11-1.31)
2500-3499	1 (Reference)	1 (Reference)	1 (Reference)
3500-4499	0.96 (0.93-0.98)	0.91 (0.88-0.94)	0.95 (0.92-0.98)
4500+	1.12 (1.02-1.22)	0.96 (0.86-1.07)	1.04 (0.94-1.15)
Year of birth, 2008-2014	0.95 (0.91-0.99)	1.10 (1.06-1.14)	1.11 (1.08-1.15)
Caesarean section	1.01 (0.98-1.04)	0.93 (0.9-0.97)	1.08 (1.04-1.11)
Season of birth			
Spring	1 (Reference)	1 (Reference)	1 (Reference)
Summer	1.33 (1.27-1.40)	1.25 (1.19-1.31)	1.13 (1.08-1.19)
Autumn	1.05 (0.99-1.11)	0.91 (0.86-0.98)	0.78 (0.74-0.84)
Winter	0.75 (0.71-0.78)	0.72 (0.69-0.76)	0.67 (0.64-0.70)
Severe neonatal comorbidity	1.05 (0.96-1.14)	1.22 (1.11-1.35)	1.21 (1.11-1.32)
Maternal education			
University	1 (Reference)	1 (Reference)	1 (Reference)

Certificate	1.27 (1.20-1.34)	1.98 (1.85-2.13)	1.83 (1.72-1.94)
Year 12	1.26 (1.17-1.36)	2.07 (1.9-2.25)	1.89 (1.76-2.04)
Below year 12	1.41 (1.32-1.51)	2.89 (2.69-3.12)	2.61 (2.45-2.79)
Missing	0.99 (0.90-1.08)	1.74 (1.58-1.91)	1.60 (1.47-1.75)
Maternal occupation			
Managers/professionals	1 (Reference)	1 (Reference)	1 (Reference)
Tradesperson	0.98 (0.93-1.04)	1.03 (0.97-1.10)	1.02 (0.96-1.08)
Other paid workers	1.03 (0.97-1.10)	1.21 (1.14-1.29)	1.17 (1.10-1.24)
Non paid workers	1.22 (1.15-1.29)	1.58 (1.48-1.68)	1.45 (1.37-1.53)
Unknown/not stated	1.24 (1.15-1.34)	1.69 (1.56-1.82)	1.44 (1.35-1.55)
Paternal education			
University	1 (Reference)	1 (Reference)	1 (Reference)
Certificate	1.08 (1.02-1.15)	1.66 (1.53-1.80)	1.80 (1.68-1.94)
Year 12	1.03 (0.95-1.13)	1.55 (1.41-1.70)	1.65 (1.51-1.80)
Below year 12	1.22 (1.14-1.32)	2.28 (2.08-2.48)	2.45 (2.27-2.65)
Missing	1.07 (0.98-1.17)	1.69 (1.53-1.87)	2.02 (1.84-2.21)
Paternal occupation			
Managers/professionals	1 (Reference)	1 (Reference)	1 (Reference)
Tradesperson	1.13 (1.07-1.19)	1.37 (1.29-1.45)	1.32 (1.25-1.39)
Other paid workers	1.36 (1.29-1.44)	1.71 (1.61-1.82)	1.53 (1.45-1.62)
Non paid workers	2.21 (2.05-2.39)	2.29 (2.14-2.46)	1.81 (1.70-1.93)
Unknown/not stated	1.57 (1.45-1.71)	2.06 (1.9-2.23)	1.72 (1.59-1.85)

SEIFA: Socio-Economic Index for Areas; HR, hazards ratio.

Appendix Table 4. Comparisons of maternal, birth and child characteristics for child development between population-based cohort and sibling cohort by infection-related hospitalization.

Maternal, birth and child characteristics	Population-based cohort (N=288,258)		Sibling cohort (N=18,006)	
	Not-hospitalised for infection (%)	Hospitalised for infection (%)	Not-hospitalised for infection (%)	Hospitalised for infection (%)
Overall	220344 (79.7)	56110 (20.3)	9003 (50.0)	9003 (50.0)
Maternal characteristics				
Maternal age at birth (years)				
<=19	6893 (3.1)	2545 (4.5)	292 (3.2)	377 (4.2)
20-24	29018 (13.2)	8994 (16)	1500 (16.7)	1547 (17.2)
25-29	60218 (27.3)	15732 (28)	2766 (30.7)	2735 (30.4)
30-34	74014 (33.6)	17837 (31.8)	2882 (32)	2893 (32.1)
35-39	41303 (18.7)	9109 (16.2)	1340 (14.9)	1263 (14)
40+	8898 (4.0)	1893 (3.4)	223 (2.5)	188 (2.1)
Maternal smoking during pregnancy				
No	195325 (88.6)	47964 (85.5)	7718 (85.7)	7715 (85.7)
Yes	25019 (11.4)	8146 (14.5)	1285 (14.3)	1288 (14.3)
Gestational or maternal diabetes				
No	207276 (94.1)	52967 (94.4)	8584 (95.3)	8579 (95.3)
Yes	13068 (5.9)	3143 (5.6)	419 (4.7)	424 (4.7)
Maternal country of birth				
Australia	153388 (69.6)	41807 (74.5)	6896 (76.6)	6896 (76.6)
Overseas	66956 (30.4)	14303 (25.5)	2107 (23.4)	2107 (23.4)
Maternal residential location				
Major city	171328 (77.8)	42368 (75.5)	6759 (75.1)	6731 (74.8)
Inner regional	37355 (17)	10249 (18.3)	1648 (18.3)	1665 (18.5)
Outer regional or more	11661 (5.3)	3493 (6.2)	596 (6.6)	607 (6.7)
SEIFA				
Q1 (most disadvantage)	40675 (18.5)	11521 (20.5)	1925 (21.4)	1955 (21.7)
Q2	43780 (19.9)	12030 (21.4)	1942 (21.6)	1932 (21.5)
Q3	48249 (21.9)	11496 (20.5)	1892 (21.0)	1884 (20.9)
Q4	40786 (18.5)	9574 (17.1)	1437 (16.0)	1456 (16.2)
Q5	46854 (21.3)	11489 (20.5)	1807 (20.1)	1776 (19.7)
Maternal education				
University	46877 (21.3)	10889 (19.4)	2039 (22.6)	1981 (22.0)
Certificate	59167 (26.9)	16102 (28.7)	2836 (31.5)	2799 (31.1)
Year 12	14612 (6.6)	3777 (6.7)	698 (7.8)	685 (7.6)
Below year 12	21016 (9.5)	6427 (11.5)	1081 (12.0)	1138 (12.6)
Unknown/not stated	78672 (35.7)	18915 (33.7)	2349 (26.1)	2400 (26.7)
Paternal education				
University	38126 (17.3)	8512 (15.2)	1585 (17.6)	1554 (17.3)
Certificate	58751 (26.7)	15511 (27.6)	2896 (32.2)	2856 (31.7)
Year 12	10936 (5)	2786 (5)	555 (6.2)	534 (5.9)
Below year 12	16205 (7.4)	4924 (8.8)	865 (9.6)	863 (9.6)
Unknown/not stated	96326 (43.7)	24377 (43.4)	3102 (34.5)	3196 (35.5)
Maternal occupation				
Managers/professionals	50840 (23.1)	12832 (22.9)	2285 (25.4)	2249 (25.0)
Tradesperson	29130 (13.2)	7792 (13.9)	1331 (14.8)	1302 (14.5)

Other paid workers	16275 (7.4)	4523 (8.1)	738 (8.2)	747 (8.3)
Non paid workers	38567 (17.5)	10066 (17.9)	2017 (22.4)	1996 (22.2)
Unknown/not stated	85532 (38.8)	20897 (37.2)	2632 (29.2)	2709 (30.1)
Paternal occupation				
Managers/professionals	60107 (27.3)	14690 (26.2)	2684 (29.8)	2707 (30.1)
Tradesperson	30318 (13.8)	7729 (13.8)	1486 (16.5)	1392 (15.5)
Other paid workers	23648 (10.7)	6555 (11.7)	1257 (14.0)	1211 (13.5)
Non paid workers	5762 (2.6)	1613 (2.9)	315 (3.5)	321 (3.6)
Unknown/not stated	100509 (45.6)	25523 (45.5)	3261 (36.2)	3372 (37.5)
Birth and child characteristics				
Sex				
Male	107005 (48.6)	30679 (54.7)	4358 (48.4)	4944 (54.9)
Female	113339 (51.4)	25431 (45.3)	4645 (51.6)	4059 (45.1)
Gestational age, week				
37-38	51759 (23.5)	14673 (26.2)	2114 (23.5)	2237 (24.8)
39-41	165676 (75.2)	40743 (72.6)	6768 (75.2)	6648 (73.8)
42+	2909 (1.3)	694 (1.2)	121 (1.3)	118 (1.3)
Birthweight (g)				
1500-2499	3365 (1.5)	922 (1.6)	107 (1.2)	124 (1.4)
2500-3499	114668 (52.0)	28604 (51.0)	4527 (50.3)	4455 (49.5)
3500-4499	98282 (44.6)	25481 (45.4)	4180 (46.4)	4230 (47.0)
4500+	4029 (1.8)	1103 (2.0)	189 (2.1)	194 (2.2)
Mode of delivery				
Vaginal	159184 (72.2)	39431 (70.3)	6658 (74)	6642 (73.8)
Caesarean section	61061 (27.7)	16660 (29.7)	2340 (26)	2361 (26.2)
Year of birth*				
2001-2007	103993 (47.2)	28329 (50.5)	4451 (49.4)	4683 (52.0)
2008-2014	116351 (52.8)	27781 (49.5)	4552 (50.6)	4320 (48.0)
Season of birth				
Spring	56318 (25.6)	13770 (24.5)	2350 (26.1)	2158 (24.0)
Summer	52844 (24.0)	13551 (24.2)	2193 (24.4)	2172 (24.1)
Autumn	55325 (25.1)	14709 (26.2)	2165 (24.0)	2418 (26.9)
Winter	55857 (25.3)	14080 (25.1)	2295 (25.5)	2255 (25.0)
Severe neonatal morbidity				
No	216954 (98.5)	54348 (96.9)	8880 (98.6)	8738 (97.1)
Yes	3390 (1.5)	1762 (3.1)	123 (1.4)	265 (2.9)
Comorbidity in childhood				
No	210618 (95.6)	49867 (88.9)	8544 (94.9)	7992 (88.8)
Yes	9726 (4.4)	6243 (11.1)	459 (5.1)	1011 (11.2)
Cumulative length of hospital stay up to 4 years, days				
0	180472 (81.9)	3519 (6.3)	7098 (78.8)	655 (7.3)
1-3	28531 (12.9)	28340 (50.5)	1385 (15.4)	4725 (52.5)
4-7	9153 (4.2)	16604 (29.6)	401 (4.5)	2500 (27.8)
8+	2188 (1.0)	7647 (13.6)	119 (1.3)	1123 (12.5)

SEIFA: Socio-Economic Index for Areas.

Appendix Table 5. Comparisons of maternal, birth and child characteristics for school performance between population-based cohort and sibling cohort by infection-related hospitalization.

Maternal, birth and child characteristics	Population-based cohort (N=667215)		Sibling cohort (N=109754)	
	Not-hospitalised for infection (%)	Hospitalised for infection (%)	Not-hospitalised for infection (%)	Hospitalised for infection (%)
Overall	509196 (79.0)	135095 (21.0)	54877 (50.0)	54877 (50.0)
Maternal characteristics				
Maternal age at birth (years)				
<=19	16573 (3.3)	6531 (4.8)	1974 (3.6)	2527 (4.6)
20-24	68302 (13.4)	22268 (16.5)	9231 (16.8)	9448 (17.2)
25-29	139787 (27.5)	38141 (28.2)	16637 (30.3)	16530 (30.1)
30-34	170812 (33.5)	42520 (31.5)	17916 (32.6)	17580 (32)
35-39	94196 (18.5)	21412 (15.8)	7969 (14.5)	7747 (14.1)
40+	19526 (3.8)	4223 (3.1)	1150 (2.1)	1045 (1.9)
Maternal smoking during pregnancy				
No	447063 (87.8)	114012 (84.4)	47173 (86.0)	47053 (85.7)
Yes	62133 (12.2)	21083 (15.6)	7704 (14.0)	7824 (14.3)
Gestational or maternal diabetes				
No	483084 (94.9)	128434 (95.1)	52623 (95.9)	52614 (95.9)
Yes	26112 (5.1)	6661 (4.9)	2254 (4.1)	2263 (4.1)
Maternal country of birth				
Australia	360342 (70.8)	102314 (75.7)	42451 (77.4)	42451 (77.4)
Overseas	148854 (29.2)	32781 (24.3)	12426 (22.6)	12426 (22.6)
Maternal residential location				
Major city	398572 (78.3)	101998 (75.5)	41496 (75.6)	41452 (75.5)
Inner regional	84403 (16.6)	24613 (18.2)	9969 (18.2)	9990 (18.2)
Outer regional or more	26221 (5.1)	8484 (6.3)	3412 (6.2)	3435 (6.3)
SEIFA				
Q1 (most disadvantage)	95935 (18.8)	28403 (21.0)	11442 (20.9)	11452 (20.9)
Q2	101040 (19.8)	28877 (21.4)	11488 (20.9)	11546 (21.0)
Q3	110410 (21.7)	27316 (20.2)	11217 (20.4)	11144 (20.3)
Q4	94886 (18.6)	23029 (17)	9273 (16.9)	9355 (17.0)
Q5	106925 (21)	27470 (20.3)	11457 (20.9)	11380 (20.7)
Maternal education				
University	155263 (30.5)	35787 (26.5)	15512 (28.3)	15397 (28.1)
Certificate	196651 (38.6)	53865 (39.9)	21997 (40.1)	21735 (39.6)
Year 12	49318 (9.7)	12735 (9.4)	5443 (9.9)	5537 (10.1)
Below year 12	71428 (14.0)	21802 (16.1)	8568 (15.6)	8644 (15.8)
Unknown/not stated	36536 (7.2)	10906 (8.1)	3357 (6.1)	3564 (6.5)
Paternal education				
University	132490 (26.0)	29372 (21.7)	12658 (23.1)	12656 (23.1)
Certificate	195732 (38.4)	51935 (38.4)	22199 (40.5)	22011 (40.1)
Year 12	37748 (7.4)	9576 (7.1)	4149 (7.6)	4175 (7.6)
Below year 12	55813 (11.0)	16736 (12.4)	6782 (12.4)	6779 (12.4)
Unknown/not stated	87413 (17.2)	27476 (20.3)	9089 (16.6)	9256 (16.9)
Maternal occupation				
Managers/professionals	170300 (33.4)	43143 (31.9)	17925 (32.7)	17809 (32.5)
Tradesperson	97099 (19.1)	26084 (19.3)	10280 (18.7)	10315 (18.8)

Other paid workers	56200 (11.0)	15127 (11.2)	5833 (10.6)	5745 (10.5)
Non paid workers	123608 (24.3)	32180 (23.8)	14582 (26.6)	14306 (26.1)
Unknown/not stated	61989 (12.2)	18561 (13.7)	6257 (11.4)	6702 (12.2)
Paternal occupation				
Managers/professionals	203370 (39.9)	49196 (36.4)	21327 (38.9)	21396 (39)
Tradesperson	100757 (19.8)	26120 (19.3)	11232 (20.5)	11070 (20.2)
Other paid workers	77121 (15.1)	21157 (15.7)	8918 (16.3)	8793 (16.0)
Non paid workers	25715 (5.1)	6719 (5.0)	2594 (4.7)	2550 (4.6)
Unknown/not stated	102233 (20.1)	31903 (23.6)	10806 (19.7)	11068 (20.2)
Birth and child characteristics				
Sex				
Male	248275 (48.8)	73927 (54.7)	26525 (48.3)	30487 (55.6)
Female	260921 (51.2)	61168 (45.3)	28352 (51.7)	24390 (44.4)
Gestational age, week				
37-38	114547 (22.5)	34033 (25.2)	12452 (22.7)	12928 (23.6)
39-41	386342 (75.9)	99112 (73.4)	41574 (75.8)	41078 (74.9)
42+	8307 (1.6)	1950 (1.4)	851 (1.6)	871 (1.6)
Birthweight (g)				
1500-2499	7858 (1.5)	2351 (1.7)	678 (1.2)	829 (1.5)
2500-3499	262408 (51.5)	68854 (51)	27305 (49.8)	27236 (49.6)
3500-4499	229337 (45)	61226 (45.3)	25759 (46.9)	25663 (46.8)
4500+	9593 (1.9)	2664 (2.0)	1135 (2.1)	1149 (2.1)
Mode of delivery				
Vaginal	374308 (73.5)	96687 (71.6)	40852 (74.4)	40725 (74.2)
Caesarean section	134688 (26.5)	38353 (28.4)	13998 (25.5)	14123 (25.7)
Year of birth*				
2001-2007	270873 (53.2)	77057 (57.0)	31148 (56.8)	32850 (59.9)
2008-2014	238323 (46.8)	58038 (43.0)	23729 (43.2)	22027 (40.1)
Season of birth				
Spring	132751 (26.1)	33880 (25.1)	14338 (26.1)	13713 (25.0)
Summer	124059 (24.4)	32906 (24.4)	13319 (24.3)	13361 (24.3)
Autumn	123805 (24.3)	34596 (25.6)	13236 (24.1)	14129 (25.7)
Winter	128581 (25.3)	33713 (25.0)	13984 (25.5)	13674 (24.9)
Severe neonatal morbidity				
No	502028 (98.6)	131036 (97.0)	54162 (98.7)	53300 (97.1)
Yes	7168 (1.4)	4059 (3.0)	715 (1.3)	1577 (2.9)
Comorbidity in childhood				
No	483095 (94.9)	118199 (87.5)	51658 (94.1)	48526 (88.4)
Yes	26101 (5.1)	16896 (12.5)	3219 (5.9)	6351 (11.6)
Cumulative length of hospital stay up to 4 years, days				
0	414184 (81.3)	8397 (6.2)	42673 (77.8)	3877 (7.1)
1-3	68062 (13.4)	67415 (49.9)	8533 (15.5)	28176 (51.3)
4-7	21369 (4.2)	40202 (29.8)	2873 (5.2)	15966 (29.1)
8+	5581 (1.1)	19081 (14.1)	798 (1.5)	6858 (12.5)

SEIFA: Socio-Economic Index for Areas.

Appendix Table 6. Associations of maternal, infant and child characteristics with child development and school performance in the sibling cohort.

Maternal, infant and child characteristics	Adjusted OR for DHR (95%CI)	Adjusted OR for numeracy (95%CI)	Adjusted OR for reading (95%CI)
Infection-related hospitalization	1.03 (0.92-1.16)	1.15 (1.07-1.24)	1.02 (0.95-1.10)
Maternal age at birth, years			
<=19	0.82 (0.41-1.61)	0.72 (0.46-1.11)	0.72 (0.48-1.09)
20-24	0.93 (0.58-1.48)	0.90 (0.66-1.22)	0.72 (0.54-0.97)
25-29	0.94 (0.70-1.27)	0.95 (0.78-1.17)	0.83 (0.69-1.01)
30-34	1 (Reference)	1 (Reference)	1 (Reference)
35-39	0.99 (0.69-1.43)	0.91 (0.7-1.19)	0.98 (0.77-1.25)
40+	1.38 (0.63-3.04)	1.22 (0.63-2.39)	2.01 (1.10-3.67)
Smoking during pregnancy	0.97 (0.69-1.35)	0.90 (0.74-1.10)	1.04 (0.86-1.26)
Maternal diabetes	1.19 (0.75-1.90)	1.25 (0.92-1.70)	0.81 (0.60-1.08)
Residence			
Major city	1 (Reference)	1 (Reference)	1 (Reference)
Inner regional	0.46 (0.26-0.81)	1.06 (0.75-1.50)	1.01 (0.73-1.42)
Outer regional or remote	0.35 (0.16-0.80)	1.20 (0.73-1.98)	1.07 (0.67-1.71)
SEIFA			
Q1 (most disadvantaged)	1.82 (0.96-3.46)	0.81 (0.50-1.32)	1.06 (0.67-1.67)
Q2	1.31 (0.70-2.46)	0.89 (0.55-1.45)	1.06 (0.68-1.66)
Q3	1.10 (0.57-2.12)	0.85 (0.51-1.42)	0.94 (0.6-1.47)
Q4	1.19 (0.66-2.14)	1.13 (0.68-1.86)	1.1 (0.67-1.81)
Q5	1 (Reference)	1 (Reference)	1 (Reference)
Male sex	3.09 (2.57-3.71)	1.19 (1.07-1.33)	2.07 (1.87-2.3)
Gestational age, week			
37-38	0.99 (0.79-1.24)	0.92 (0.8-1.05)	0.99 (0.86-1.13)
39-41	1 (Reference)	1 (Reference)	1 (Reference)
42+	1.99 (0.87-4.56)	0.91 (0.6-1.38)	1.21 (0.84-1.73)
Birthweight, gram			
1500-2499	1.64 (0.86-3.12)	1.15 (0.83-1.59)	1.41 (0.97-2.06)
2500-3499	1 (Reference)	1 (Reference)	1 (Reference)
3500-4499	0.95 (0.76-1.18)	0.96 (0.84-1.10)	0.83 (0.73-0.94)
4500+	0.66 (0.37-1.19)	0.96 (0.61-1.50)	0.69 (0.45-1.05)
Year of birth, 2008-2014	0.99 (0.77-1.28)	0.93 (0.79-1.08)	0.81 (0.7-0.94)
Caesarean section	1.29 (0.88-1.90)	1.00 (0.78-1.27)	0.80 (0.63-1.01)
Season of birth			
Spring	1 (Reference)	1 (Reference)	1 (Reference)
Summer	1.22 (0.95-1.55)	1.25 (1.08-1.45)	1.26 (1.10-1.46)
Autumn	1.19 (0.94-1.52)	1.15 (0.99-1.33)	1.23 (1.07-1.41)
Winter	0.87 (0.68-1.12)	1.11 (0.96-1.29)	1.00 (0.87-1.15)
Younger sibling	1.02 (0.82-1.26)	0.96 (0.86-1.08)	0.78 (0.70-0.87)
Severe neonatal comorbidity	1.09 (0.64-1.86)	1.23 (0.87-1.72)	1.19 (0.87-1.61)
Maternal occupation			
Managers/professionals	1 (Reference)	1 (Reference)	1 (Reference)
Tradesperson	0.84 (0.53-1.34)	1.19 (0.86-1.65)	0.99 (0.72-1.35)
Other paid workers	0.80 (0.49-1.31)	1.01 (0.72-1.42)	0.94 (0.69-1.30)
Non paid workers	1.17 (0.77-1.79)	1.28 (0.93-1.75)	1.30 (0.96-1.76)
Unknown/not stated	1.33 (0.86-2.05)	1.05 (0.76-1.45)	1.16 (0.85-1.57)
Paternal occupation			
Managers/professionals	1 (Reference)	1 (Reference)	1 (Reference)
Tradesperson	0.80 (0.51-1.25)	1.33 (0.96-1.84)	1.11 (0.83-1.49)
Other paid workers	0.92 (0.59-1.44)	1.20 (0.87-1.66)	1.11 (0.83-1.49)
Non paid workers	1.78 (1.03-3.05)	1.17 (0.82-1.66)	0.81 (0.58-1.12)
Unknown/not stated	0.87 (0.58-1.31)	1.31 (0.97-1.76)	1.14 (0.87-1.50)

DHR: development high-risk; SEIFA: Socio-Economic Index for Areas; aOR, adjusted odds ratio.

Appendix Table 7. Stratified analysis for child development and school performance from sibling cohort.

	DHR			Numeracy			Reading		
	**Cases/exposed**	**OR**	**Adjusted OR***	**Cases/exposed**	**OR**	**Adjusted OR***	**Cases/exposed**	**OR**	**Adjusted OR***
SEIFA									
Q1 (most disadvantaged)	314/1955	1.23 (0.98-1.54)	1.24 (0.96-1.59)	669/11349	1.05 (0.92-1.21)	1.05 (0.91-1.20)	788/11400	1.15 (1.02-1.3)	1.12 (0.98-1.27)
Q2	248/1932	1.24 (0.97-1.57)	1.20 (0.91-1.57)	535/11467	1.24 (1.07-1.44)	1.24 (1.06-1.45)	622/11499	1.14 (0.99-1.31)	1.07 (0.92-1.24)
Q3	171/1884	0.83 (0.63-1.09)	0.73 (0.54-1.00)	411/11052	1.25 (1.05-1.48)	1.23 (1.03-1.46)	459/11094	0.92 (0.79-1.07)	0.84 (0.71-0.99)
Q4	114/1456	1.05 (0.73-1.53)	1.06 (0.66-1.71)	197/9300	1.08 (0.83-1.4)	1.04 (0.79-1.36)	241/9326	1.08 (0.87-1.33)	1.02 (0.80-1.29)
Q5	94/1776	0.92 (0.66-1.28)	0.81 (0.53-1.22)	120/11333	1.42 (1.03-1.94)	1.29 (0.91-1.84)	137/11360	0.99 (0.75-1.3)	0.97 (0.71-1.33)
Q1 and Q2	562/3887	1.25 (1.07-1.45)	**1.18 (1.00-1.4)**	1204/22816	1.13 (1.03-1.24)	**1.12 (1.02-1.24)**	1410/22899	1.14 (1.04-1.24)	1.07 (0.98-1.18)
Maternal education									
Certificate or higher	362/4780	1.05 (0.85-1.28)	0.92 (0.73-1.16)	798/36941	1.23 (1.1-1.39)	1.23 (1.09-1.38)	965/37037	1.07 (0.96-1.18)	1.02 (0.91-1.14)
Year 12 or below	292/1823	1.13 (0.87-1.46)	1.04 (0.77-1.4)	932/14038	1.18 (1.05-1.32)	1.18 (1.04-1.32)	1041/14106	1.05 (0.94-1.17)	0.99 (0.88-1.11)
Maternal occupation									
Manager or professionals	111/2249	0.98 (0.67-1.44)	0.99 (0.62-1.58)	230/17740	1.53 (1.21-1.95)	1.52 (1.19-1.95)	265/17768	0.85 (0.7-1.04)	0.83 (0.67-1.03)
Tradesperson	105/1302	1.39 (0.90-2.13)	1.33 (0.78-2.29)	239/10256	1.03 (0.81-1.31)	1.02 (0.79-1.32)	309/10295	1.15 (0.93-1.41)	1.10 (0.89-1.37)
Other paid workers	71/747	0.87 (0.48-1.58)	0.87 (0.46-1.66)	258/5693	1.17 (0.93-1.48)	1.20 (0.94-1.53)	285/5722	1.03 (0.83-1.29)	0.96 (0.76-1.21)
Non-paid workers	312/1996	1.14 (0.87-1.48)	1.15 (0.86-1.56)	811/14183	1.28 (1.12-1.46)	1.28 (1.12-1.47)	945/14233	1.22 (1.08-1.38)	1.16 (1.02-1.31)
Paternal education									
Certificate or higher	311/4410	1.04 (0.83-1.30)	0.90 (0.69-1.18)	714/34496	1.31 (1.15-1.48)	1.31 (1.15-1.49)	851/34579	1.03 (0.92-1.15)	0.97 (0.86-1.09)
Year 12 or below	190/1397	1.01 (0.74-1.39)	1.03 (0.71-1.48)	602/10853	1.06 (0.91-1.24)	1.03 (0.88-1.21)	687/10904	1.08 (0.94-1.24)	1.00 (0.87-1.16)
Paternal occupation									
Manager or professionals	132/2707	0.78 (0.56-1.09)	0.63 (0.41-0.96)	232/21307	1.15 (0.92-1.44)	1.15 (0.91-1.44)	288/21354	0.95 (0.78-1.15)	0.90 (0.73-1.11)
Tradesperson	109/1392	1.58 (1.02-2.44)	1.49 (0.9-2.46)	310/11001	1.23 (1.01-1.51)	1.21 (0.98-1.49)	417/11031	1.21 (1.01-1.44)	1.11 (0.92-1.34)
Other paid workers	159/1211	1.21 (0.85-1.73)	1.21 (0.83-1.76)	437/8738	1.13 (0.95-1.35)	1.11 (0.92-1.32)	509/8755	1.16 (0.99-1.37)	1.07 (0.90-1.27)
Non-paid workers	75/321	1.00 (0.50-2.00)	1.16 (0.54-2.49)	213/2512	1.23 (0.90-1.67)	1.18 (0.87-1.62)	207/2541	1.04 (0.76-1.41)	1.03 (0.76-1.41)
Sex									
Male	684/4944	0.98 (0.82-1.18)	1.00 (0.83-1.21)	1148/30291	1.21 (1.06-1.38)	1.23 (1.07-1.41)	1557/30354	0.97 (0.86-1.08)	0.98 (0.87-1.10)
Female	257/4059	1.13 (0.86-1.48)	1.21 (0.90-1.64)	784/24210	1.1 (0.94-1.27)	1.09 (0.93-1.27)	690/24325	1.02 (0.88-1.19)	1.02 (0.87-1.19)

*Adjusted for maternal age, maternal smoking, Socio-Economic Index for Areas (SEIFA), maternal education and occupation, paternal education and occupation, and child sex. However, only maternal age, maternal smoking, and child sex were adjusted for the stratified analysis by occupation. DHR: development high-risk; SEIFA: Socio-Economic Index for Areas; aOR, adjusted odds ratio.

Appendix Table 8. Associations of infection-related hospitalization with child development and school performance by different categories of infection in the sibling cohort.

	DHR			Numeracy			Reading		
	Cases/ exposed	OR (95%CI)	Adjusted OR* 95%CI	Cases/ exposed	OR (95%CI)	Adjusted OR* 95%CI	Cases/ exposed	OR (95%CI)	Adjusted OR* 95%CI
Organ specific infection									
Gastrointestinal infections	140/1448	1.05 (0.80-1.39)	1.10 (0.78-1.55)	355/10039	1.04 (0.88-1.22)	1.06 (0.89-1.26)	420/10084	0.99 (0.85-1.15)	1.02 (0.87-1.21)
Skin infection	64/462	1.31 (0.85-2.04)	1.69 (0.85-3.35)	127/2595	1.21 (0.91-1.62)	1.31 (0.95-1.80)	131/2614	1.13 (0.86-1.49)	1.10 (0.82-1.48)
Urinary tract infection	40/490	1.07 (0.64-1.77)	1.06 (0.53-2.12)	80/2662	1.31 (0.93-1.84)	1.47 (0.96-2.24)	98/2666	1.35 (0.98-1.85)	1.47 (0.98-2.21)
Upper respiratory tract infection	241/2560	1.06 (0.86-1.31)	0.95 (0.74-1.21)	495/15343	1.17 (1.01-1.34)	1.14 (0.99-1.32)	576/15368	1.02 (0.90-1.15)	0.94 (0.82-1.07)
Lower respiratory tract infection	415/3334	1.26 (1.07-1.48)	1.10 (0.91-1.32)	810/19021	1.20 (1.07-1.34)	1.18 (1.05-1.33)	960/19102	1.12 (1.01-1.23)	1.04 (0.93-1.15)
Vaccine-preventable infection	46/372	1.32 (0.79-2.22)	1.64 (0.62-4.32)	103/2658	0.86 (0.65-1.15)	0.84 (0.60-1.16)	117/2664	0.92 (0.70-1.22)	0.78 (0.56-1.10)
Number of infections									
1	744/7227	1.09 (0.96-1.22)	0.99 (0.87-1.13)	1483/43743	1.16 (1.07-1.26)	1.16 (1.07-1.26)	1698/43872	1.06 (0.99-1.14)	1.01 (0.93-1.08)
2	145/1350	1.35 (1.02-1.80)	1.40 (1.00-1.96)	313/8237	1.14 (0.95-1.36)	1.13 (0.94-1.37)	399/8274	1.06 (0.91-1.24)	0.99 (0.84-1.17)
3+	52/426	1.14 (0.73-1.80)	0.94 (0.53-1.65)	136/2521	1.22 (0.92-1.62)	1.15 (0.85-1.55)	150/2533	1.22 (0.95-1.56)	1.18 (0.90-1.55)
Age at first infection, year									
<1	498/4349	1.13 (0.97-1.30)	1.10 (0.78-1.55)	993/25866	1.17 (1.06-1.30)	1.18 (1.07-1.31)	1185/25953	1.09 (0.99-1.19)	1.03 (0.94-1.13)
1-2	208/2373	1.02 (0.82-1.27)	0.94 (0.73-1.21)	470/14263	1.16 (1.01-1.34)	1.17 (1.01-1.36)	535/14297	1.05 (0.93-1.20)	1.03 (0.90-1.19)
2-3	135/1270	1.17 (0.87-1.56)	1.22 (0.84-1.77)	274/7961	1.29 (1.06-1.57)	1.23 (1.00-1.51)	282/8004	1.00 (0.84-1.19)	0.91 (0.75-1.10)
3-4	100/1011	1.32 (0.94-1.85)	1.55 (0.98-2.44)	195/6411	0.97 (0.78-1.21)	0.96 (0.76-1.21)	245/6425	1.14 (0.94-1.38)	1.08 (0.87-1.34)
Length of hospitalised for infection, day									
<=1	441/4614	1.06 (0.91-1.24)	0.95 (0.81-1.13)	962/27742	1.20 (1.08-1.33)	1.19 (1.07-1.32)	1096/27846	1.02 (0.93-1.12)	0.95 (0.87-1.05)
2	218/2044	1.10 (0.88-1.38)	1.06 (0.83-1.37)	438/11875	1.09 (0.94-1.27)	1.05 (0.90-1.23)	524/11916	1.07 (0.94-1.22)	1.03 (0.89-1.19)
3+	282/2345	1.26 (1.03-1.54)	1.12 (0.89-1.42)	532/14884	1.16 (1.01-1.32)	1.17 (1.02-1.35)	627/14917	1.17 (1.03-1.32)	1.12 (0.99-1.28)

*Adjusted for maternal age, maternal smoking, Socio-Economic Index for Areas (SEIFA), maternal education and occupation, paternal education and occupation, and child sex. DHR: development high-risk; SEIFA: Socio-Economic Index for Areas; OR, odds ratio

Appendix Table 9. Top 3 diagnosis for each organ-specific infections from the cohort for child development and school performance.

Description of diagnosis	ICD10-AM code	Cohort for development n/ N (%)	Cohort for numeracy n/ N (%)	Cohort for reading n/ N (%)
Gastrointestinal infection				
Viral and other specified intestinal infections	A08	5287/9710 (54.4)	14113/25767 (54.8)	14149/25867 (54.7)
Other gastroenteritis and colitis of infectious and unspecified origin	A09	3975/9710 (40.9)	10637/25767 (41.3)	10693/25867 (41.3)
Other salmonella infections	A02	245/9710 (2.5)	520/25767 (2.0)	522/25867 (2.0)
Skin infection				
Cellulitis of face	L032	629/2832 (22.2)	1480/6406 (23.1)	1492/6458 (23.1)
Cellulitis of lower limb	L0311	316/2832 (11.2)	791/6406 (12.3)	795/6458 (12.3)
Cutaneous abscess of limb	L024	186/2832 (6.6)	488/6406 (7.6)	492/6458 (7.6)
Urinary tract infection (UTI)				
UTI, site not specified	N39	2803/3012 (93.1)	6294/6751 (93.2)	6319/6776 (93.3)
Neonatal UTI	P39	131/3012 (4.3)	301/6751 (4.5)	301/6776 (4.4)
Orchitis, epididymitis and epididymo-orchitis without abscess	N45	37/3012 (1.2)	75/6751 (1.1)	74/6776 (1.1)
Upper respiratory tract infection				
Acute upper respiratory infections of multiple and unspecified sites	J06	5065/17069 (29.7)	12349/40180 (30.7)	12371/40272 (30.7)
Nonsuppurative otitis media	H65	4836/17069 (28.3)	10920/40180 (27.2)	10946/40272 (27.2)
Suppurative and unspecified otitis media	H66	2369/17069 (13.9)	5640/40180 (14.0)	5650/40272 (14.0)
Lower respiratory tract infection				
Acute bronchitis	J21	10489/19974 (52.5)	23615/46616 (50.7)	23714/46793 (50.7)
Acute obstructive laryngitis	J05	3070/19974 (15.4)	7610/46616 (16.3)	7633/46793 (16.3)
Pneumonia, organism unspecified	J18	2799/19974 (14.0)	7385/46616 (15.8)	7411/46793 (15.8)
Central nervous system infection				
Viral meningitis	A87	285/620 (46.0)	517/1289 (40.1)	515/1290 (39.9)
Meningitis due to other and unspecified causes	G03	96/620 (15.5)	233/1289 (18.1)	236/1290 (18.3)
Meningococcal infection	A39	82/620 (13.2)	175/1289 (13.6)	174/1290 (13.5)
Vaccine preventable disease				
Rotaviral enteritis	A080	1349/2516 (53.6)	3891/6763 (57.5)	3898/6785 (57.5)
Influenza	J10	503/2516 (20.0)	1099/6763 (16.3)	1108/6785 (16.3)
Whooping cough	A37	275/2516 (10.9)	801/6763 (11.8)	803/6785 (11.8)
